# What Platform Scores Miss: Multidimensional Evaluation of AI Teaching Agents in Medical Education

**DOI:** 10.2196/96819

**Published:** 2026-09-03

**Authors:** Hui Zhang, Lihui Qu, Jianmin Zheng, Yi Xiong, Hongbo Bai, Ruiying Ji, Guohui Liu, Wanling Chen, Zirui Cheng, Youbang Chen, Chun-tao Yang

**Affiliations:** 1 Office of Academic Affairs, Guangzhou Medical University, Guangzhou, China; 2 Department of Physiology, School of Basic Medical Sciences, The Fourth Affiliated Hospital of Guangzhou Medical University, Guangzhou, China; 3 Guangzhou Municipal and Guangdong Provincial Key Laboratory of Protein Modification and Disease, Department of Physiology, School of Basic Medical Sciences, Guangzhou Medical University, 1 Xinzao Road, Guangzhou, Guangdong, 511436, China, 86 20-37103213; 4 Department of Endocrinology, The First Affiliated Hospital of Guangzhou Medical University, Guangzhou, China; 5 Zhongshan School of Medicine, Sun Yat-Sen University, Guangzhou, China

**Keywords:** AI teaching agents, educational data science, educational evaluation, endocrinology, large language models, LLMs, multidimensional rubric, precision education, role-play

## Abstract

**Background:**

Large language model (LLM)–based AI teaching agents are increasingly used in medical education, yet their pedagogical quality is typically judged by platform-generated scores whose scoring criteria are undisclosed and may not reflect the teaching quality of the agent.

**Objective:**

This study aimed to develop and validate a multidimensional rubric for evaluating AI teaching agents and to examine the correspondence between platform scores and rubric-based teaching quality.

**Methods:**

Eight AI teaching agents covering an endocrinology curriculum were deployed across 4 role-play paradigms (patient, student, expert, and family). Twenty-two fourth-year medical students generated 167 dialogues, which were scored both by the platform and by an independently applied 8-dimension rubric (100 points, covering knowledge accuracy, pedagogical guidance, knowledge coverage, role-play quality, difficulty calibration, medical safety, student engagement, and feedback quality). Each dialogue was scored 4 times by a primary evaluator (Claude Opus 4.8; mean within-model SD 0.36), with 2 additional LLMs as robustness checks; 40 dialogues spanning all agents were rescored by a medical-education expert for validation.

**Results:**

Platform and rubric rankings diverged for most agents: the agent ranked third by the platform ranked last on rubric-based quality, and the platform’s fourth-ranked agent ranked first. Agents differed most on knowledge-related dimensions (knowledge coverage coefficient of variation=27.3%) and least on role-play quality (coefficient of variation=5.7%), while difficulty calibration was a shared weakness. In a case-level observation, one agent revised specifically to strengthen empathy attained high role-play quality yet the lowest knowledge coverage of all agents. AI scores agreed with expert ratings at the total-score level (intraclass correlation coefficient=0.51) and on cognitive-process dimensions, but agreement was low for the more subjective dimensions. Student gender showed no detectable effect, though this analysis was underpowered.

**Conclusions:**

In this exploratory study, platform-generated scores reflected a construct different from agent teaching quality and should be used as a complement rather than as the sole quality indicator. The 8-dimension rubric provides a transparent, standardized alternative that reveals differences missed by platform scores, including a lack of association between empathy and knowledge coverage that warrants attention in future agent design.

## Introduction

Bridging the gap between theoretical knowledge and clinical competence remains a central challenge in medical education, particularly in disciplines requiring multisystem integration such as endocrinology [1,2]. Simulated patient encounters are among the most effective approaches for developing clinical reasoning and communication skills, but their scalability is constrained by the cost and limited availability of standardized human patients [3-7]. Large language model (LLM)–based AI agents have emerged as a promising alternative, achieving clinical fidelity comparable to standardized human patients at substantially lower cost [8-13], and they are now being adopted in medical curricula at a pace that outstrips our ability to evaluate them. Yet nearly all existing implementations adopt a single role-play paradigm in which the AI portrays a patient and the student acts as a physician, without examining whether alternative role configurations produce different teaching outcomes, or how the quality of any such agent should be judged in the first place.

These developments expose a more fundamental gap: we lack a validated way to judge whether an AI teaching agent teaches well. Existing evaluation frameworks address clinical intervention outcomes or clinical task accuracy [14-16] but not the teaching-specific dimensions that determine educational value, such as adaptive difficulty calibration, knowledge coverage, and formative feedback quality. In practice, quality is often inferred from the aggregate scores generated by commercial teaching platforms, yet these scores are produced by undisclosed criteria that cannot be independently verified, are not decomposable by dimension, and are not comparable across agents, leaving it unclear what they actually measure and whether they reflect the teaching quality of the agent or merely the surface characteristics of a student’s responses. This evaluation gap has a direct consequence: without a trustworthy measure of teaching quality, the design variables that might improve these agents, including role-play configuration, content domain, and learner characteristics, such as gender, cannot be studied systematically and remain largely unexplored within a single controlled context [6,17].

Endocrinology is well suited to investigating these questions, spanning molecular mechanisms to chronic disease management within a single curricular module, yet remaining heavily reliant on traditional didactics, with simulation-based approaches underrepresented [18-20]. Against this background, we deployed 8 AI teaching agents covering a complete endocrinology curriculum, each employing a distinct role-play paradigm, and analyzed 167 student-agent dialogues. Because the platform’s built-in scores proved uninterpretable as measures of teaching quality, we developed a purpose-built 8-dimension rubric, validated against multiple independent LLM evaluators and a human expert, as a transparent external benchmark. To our knowledge, this is the first study to (1) propose and validate a multidimensional framework for evaluating AI teaching agents in medical education, (2) examine how role-play design, content domain, and learner gender relate to teaching quality within a unified curriculum, and (3) compare platform-generated scores against a transparent external standard to clarify what each actually captures. Beyond the specific agents studied, this work offers a reusable approach to a problem the field will increasingly face: how to see what an AI teaching agent is actually teaching.

## Methods

### Study Design and Participants

This cross-sectional observational study was conducted during the autumn semester of 2025 at Guangzhou Medical University with 22 fourth-year undergraduate students majoring in basic medical sciences, who participated as part of their endocrinology curriculum. The cohort comprised 15 female and 7 male students. Each student interacted with all 8 AI teaching agents sequentially over the course of the semester. Owing to individual absences, the number of completed dialogues per agent ranged from 19 to 22, yielding 167 valid dialogues in total.

### Ethical Considerations

All participants were informed of the study purpose, and the study was approved by the institutional review board of Guangzhou Medical University (202607001).

### AI Teaching Agent Design and Deployment

Eight AI teaching agents were constructed and deployed on the Chaoxing e-Learning platform (Beijing Century Chaoxing Information Technology Development Co, Ltd), a widely used commercial platform in Chinese higher education. Each agent covered 1 chapter of the endocrinology curriculum. Two chapters addressed foundational content (a general introduction and organ morphology), and 6 addressed clinical or disease-oriented content (hypothalamic-pituitary diseases, thyroid diseases, adrenal diseases, glucose metabolism disorders, lipid metabolism disorders, and calcium-phosphorus metabolism disorders).

Each agent employed a distinct role-play scenario assigned to 1 of 4 role paradigms: patient (A1, A4, A5), student (A2, A3), expert (A6, A7), and family member (A8); full configurations are provided in Table 1. The 4 paradigms were chosen to represent 4 prototypical interaction scenarios in clinical medicine (doctor and patient, teacher and student, expert consultation, and family communication), thereby sampling a range of communicative and pedagogical demands rather than instantiating a single learning theory a priori. Where relevant, the resulting behaviors could be related post hoc to established educational concepts; for example, the student paradigm elicits scaffolding within the learner’s zone of proximal development [20,21]. We emphasize that because each agent combined 1 role paradigm with 1 content chapter, role and content were confounded by design.

Key implementation differences among the agents lay in the assigned AI persona and the reciprocal role assigned to the student, which together determined the communicative register and the pedagogical stance of each dialogue (Table 1). For example, within the expert paradigm, agent A6 positioned the student as a learner in a mentorship model, whereas agent A7 positioned the student as a patient, providing both a physician and a patient perspective. Agent A8 was deliberately revised after the initial deployment of the first 7 agents, when their empathic engagement was judged to be limited; only its prompt was modified, while all other settings were held constant, to strengthen an empathic family-member persona. For each agent, the research team specified the instructional content, learning objectives, knowledge sources, role-play scenario, and safety and behavioral constraints; the complete per-agent configuration, including prompts, is provided in Multimedia Appendix 1.

**Table 1.** Overview of AI teaching agent configurations.

Agent	Content topic	Role paradigm	AI role	Student role
A1	General introduction to endocrinology	Patient	Patient (endocrinology inpatient)	Physician (endocrinology intern)
A2	Organ morphology	Student	Learner (first-year medical student)	Instructor (anatomy and histology teacher)
A3	Hypothalamic-pituitary diseases	Student	Learner (average-performing student)	Instructor (top-performing class representative)
A4	Thyroid diseases	Patient	Patient (inquisitive, detail-seeking patient)	Physician (patient’s physician)
A5	Adrenal diseases	Patient	Patient (Cushing syndrome patient)	Physician (endocrinologist)
A6	Glucose metabolism disorders	Expert	Expert (senior diabetes clinician-researcher)	Learner (basic medical science student)
A7	Lipid metabolism disorders	Expert	Expert (attending physician)	Patient (patient with obesity)
A8	Calcium-phosphorus metabolism disorders	Family	Family member (older patient with osteoporosis)	Caregiver (grandchild)

### Data Collection

Two types of data were collected from each student-agent dialogue. First, the platform’s built-in scoring system generated a single aggregate score per dialogue as an automated assessment of student performance. The platform does not disclose how these scores are generated, and its scoring mechanism could not be independently obtained or verified; only aggregate scores were available to instructors, with no dimensional breakdown. Second, complete dialogue transcripts were exported from the platform for independent external evaluation.

### Eight-Dimension Pedagogical Evaluation Rubric

To enable standardized cross-agent comparison of teaching quality, we developed an 8-dimension evaluation rubric (Table 2) informed by existing frameworks for AI conversational agents in health care [14], AI agent evaluation in clinical medicine [16], and educational agent effectiveness research [17], supplemented by expert discussion within the research team. Unlike platform-generated scoring, the rubric was designed to assess agent teaching quality rather than student response quality. The complete scoring prompt is provided in Multimedia Appendix 2.

Dimension weights were assigned by the research team on conceptual grounds: medical knowledge accuracy and pedagogical guidance, as the core teaching functions, received the highest weight (20 points each), whereas student engagement elicitation and feedback quality, as supportive dimensions, received the lowest (5 points each). We note as a limitation that these weights were set without formal student or educator input, and that empirically derived weights are a direction for future work.

**Table 2.** Eight-dimension pedagogical evaluation rubric for AI teaching agents.

Dimension	Points (total=100)	Scoring focus
Medical knowledge accuracy	20	Factual correctness and absence of medical errors
Pedagogical guidance ability	20	Use of questioning, scaffolding, and structured guidance
Knowledge-point coverage efficiency	15	Breadth and completeness of curriculum content addressed
Role-play quality	15	Consistency, naturalness, and appropriateness of role portrayal
Adaptive difficulty calibration	10	Adjustment of complexity in response to student level
Medical safety boundary	10	Avoidance of harmful advice and appropriate referral behavior
Student engagement elicitation	5	Prompting of active student participation and critical thinking
Assessment and feedback quality	5	Specificity and constructiveness of formative feedback

### Rubric-Based Scoring and Validation

All 167 dialogue transcripts were scored across the 8 dimensions using LLMs operating under standardized instructions [15]. To assess the robustness and reliability of the scoring, 3 LLMs from different developers were used as evaluators: Claude Opus 4.8 (Anthropic), representing a leading international model, and DeepSeek (DeepSeek V4 Artificial Intelligence Basic Technology Research Co, Ltd) and Qwen 2.5 (Alibaba Cloud), two widely used Chinese LLMs selected for their strong Chinese-language capability given that all dialogues used Chinese. DeepSeek and Qwen were added at the recommendation of a reviewer and on the basis of our recent study of mainstream domestic and international LLMs for medical assessment tasks [22].

All scoring was performed programmatically through each model’s application programming interface under a fixed request configuration, with an intercall interval of 1.0 second and up to 3 retries per call. To promote deterministic, reproducible outputs, DeepSeek and Qwen were queried with temperatures of 0 and top-p of 0.01; Claude Opus 4.8 was queried at its default setting. Each model independently applied the identical 8-dimension rubric and scored every dialogue 4 times, and the mean of the 4 runs was used as that model’s final score. This repeated-scoring design followed directly from our recent finding that the reliability of LLM-based scoring is better captured by repeated runs and cross-run variance than by single-run performance [22]. Within-model reproducibility across the 4 runs was quantified by the SD of the repeated scores; reproducibility was high, indicating that scores were stable rather than single, unrepeatable judgments [23].

To calibrate the AI-based scoring against human judgment, a medical-education expert independently rescored a stratified subset of 40 dialogues using the identical 8-dimension rubric. The subset was stratified by Claude total score to span high, medium, and low quality and to cover all 8 agents, and the expert was blinded to the AI scores. Agreement between AI and expert ratings was quantified by the Pearson correlation and the intraclass correlation coefficient (ICC), computed at the total-score level and per dimension [24]. Because rescoring was performed by a single expert, expert-to-expert reliability could not be computed; this is addressed in the Limitations section.

### Statistical Analysis

Given nonnormal score distributions and heterogeneous variances across agents (Shapiro-Wilk and Levene tests), nonparametric methods were used throughout. Platform-generated scores were compared across agents using the Kruskal-Wallis *H* test, with post hoc Dunn tests and Bonferroni correction for pairwise comparisons. Effect sizes were reported as η^2^ for omnibus tests and Cohen *d* for selected contrasts. Between-agent variation in each rubric dimension was summarized using the coefficient of variation (CV).

Comparisons across role paradigms were treated as descriptive, because the independent unit was the agent and each role group comprised only 1 to 3 agents; formal inferential testing was therefore not performed at the agent level. The divergence between platform-based and rubric-based agent rankings was characterized through concrete rank comparisons rather than a single agent-level correlation coefficient, which would be unstable at this sample size. Learner gender differences were examined across dimensions using Mann-Whitney *U* tests, with results interpreted in light of the small and imbalanced sample. The correspondence between rubric-based agent quality and students’ end-of-course chapter examination performance was examined descriptively. All analyses were conducted in Python (version 3.11), with a 2-sided significance threshold of *P*<.05.

## Results

### Platform-Generated Scores Varied Across Agents

**Figure 1.**
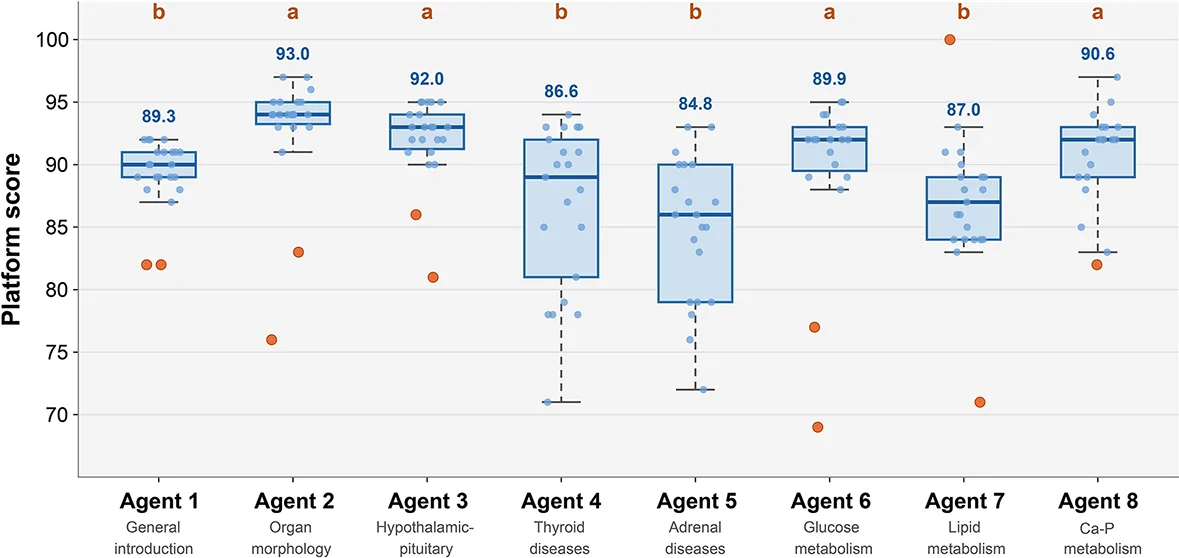
Distribution of Chaoxing platform–generated scores across 8 agents (22 students; 167 valid dialogues). Box plots show median, IQR, and 1.5×IQR whiskers; individual scores are jittered dots; outliers (>1.5×IQR) are in orange. Blue numerals above boxes denote means. Agents not sharing a common letter (a vs b) differ significantly ( Bonferroni-corrected *P*<.05).

Chaoxing platform–generated scores from 167 student-agent dialogues differed significantly across the 8 agents (Kruskal-Wallis *H*_7_=57.981; *P*<.001; η^2^=0.321; Figure 1), with 9 significant pairwise differences identified by post hoc Dunn tests (Bonferroni-corrected *P*<.05). Basic science agents (A2, A3) received the highest scores, while clinical disease agents (A4, A5, A7) received the lowest. While these differences indicate that agent design influenced student performance under platform scoring, the platform does not disclose how its scores are generated, and its scoring mechanism could not be independently verified, precluding any interpretation of what drove them or whether they reflect genuine agent teaching quality. Therefore, the 8-dimension rubric was applied as an independent external audit, reported in the following sections.

### LLM Evaluators Differed in Scoring Discrimination

To evaluate the robustness of the rubric-based scoring, the 8-dimension rubric was independently applied by 3 LLM evaluators from different developers (Claude, DeepSeek, and Qwen), each scoring all 167 dialogues 4 times, with high within-model reproducibility (Table 3; mean within-model SD≤0.36).

**Table 3.** Comparison of 3 LLM evaluators on the 8-dimension rubric.

Metric	Claude (Opus 4.8)	DeepSeek V4	Qwen 2.5
Dialogues scored (×4 runs), n	167	167	167
Intrarater SD (score stability)^a^	0.36	0.06	0.17
Agent-total score range	61‐92	82‐95	75‐100
Agent-total SD (discrimination)^b^	11.6	4.0	7.3
Overall ceiling rate^c^, %	17	30	51
Mean achievement rate, %	82	90	92
Rank correlation with Claude (ρ)^d^	—^e^	0.64	0.71

a Intrarater SD: mean within-model SD across the 4 runs (lower=more stable).

b Agent-total SD: SD of the 8 agent-level totals (higher=better discrimination).

c Overall ceiling rate: proportion of dialogue×dimension cells scored at the maximum (higher=stronger ceiling effect).

d Rank correlation: Spearman ρ of agent rankings with Claude (both *P*<.05).

e Not applicable.

**Figure 2.**
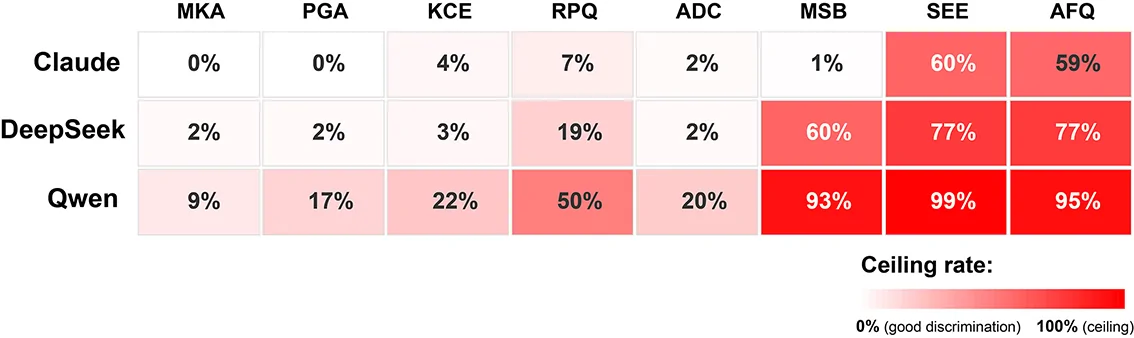
Ceiling effects across the 3 large language model evaluators on the 8 scoring dimensions. Each cell shows the proportion of the 167 dialogues scored at the maximum for that dimension; darker red indicates a stronger ceiling effect (less discrimination). ADC: adaptive difficulty calibration; AFQ: assessment and feedback quality; KCE: knowledge-point coverage efficiency; MKA: medical knowledge accuracy; MSB: medical safety boundary; PGA: pedagogical guidance ability; RPQ: role-play quality; SEE: student engagement elicitation.

The 3 models agreed broadly on the relative ranking of agents (Spearman ρ=0.64 and 0.71 for Claude vs DeepSeek and Qwen) but differed substantially in discrimination. DeepSeek and Qwen showed pronounced leniency, scoring large proportions of dialogues at the ceiling (Figure 2; Qwen: above 90% on medical safety boundary, student engagement elicitation, and assessment and feedback quality; DeepSeek: 60-77% on the same dimensions). This compressed between-agent differences, yielding narrow score ranges (82‐95 and 75‐100) and low discrimination (SD of agent total scores was 4.0 and 7.3). Claude showed a minimal ceiling effects except on the two 5-point dimensions (student engagement elicitation, assessment and feedback quality), with the widest range (61-92) and greatest discrimination (SD 11.6).

Because the analysis aimed to resolve differences in teaching quality among agents, an evaluator able to discriminate between them was required; Claude was therefore used as the primary evaluator, with its agreement against human expert judgment examined separately. The leniency of the other evaluators is itself a relevant finding, indicating that ceiling effects should be anticipated when LLMs serve as evaluators of educational quality.

### Agents Differed Mainly in Knowledge, Not in Role Performance

**Figure 3.**
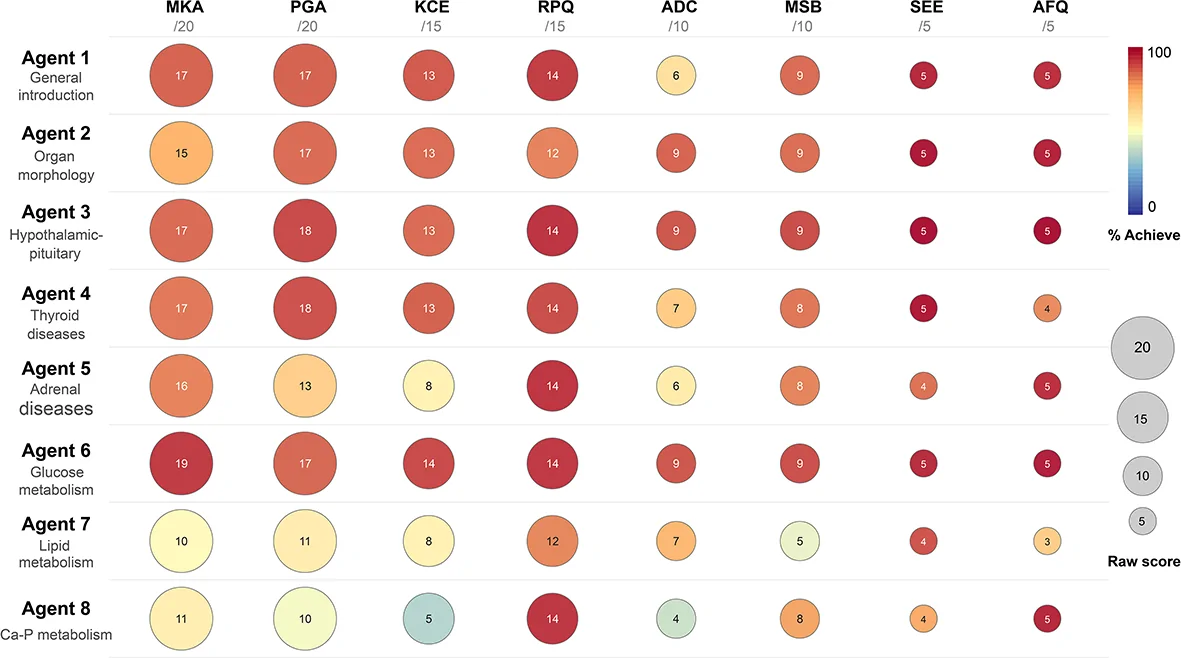
Eight-dimension rubric profiles of the 8 AI teaching agents. Scores are the mean of 4 independent runs by Claude Opus 4.8. Bubble size indicates the dimension's maximum score, color indicates the achievement rate; and number indicates the raw score. ADC: adaptive difficulty calibration; AFQ: assessment and feedback quality; KCE: knowledge-point coverage efficiency; MKA: medical knowledge accuracy; MSB: medical safety boundary; PGA: pedagogical guidance ability; RPQ: role-play quality; SEE: student engagement elicitation.

Applying the 8-dimension rubric, agent total scores ranged widely (60.6‐91.6; Figure 3), with A6 and A3 scoring highest and A7 and A8 lowest. This variation was not uniform across dimensions. It was largest on the 2 knowledge-related dimensions, namely knowledge-point coverage (CV=27.3%) and medical knowledge accuracy (CV=18.5%), indicating that agents differed most in what and how accurately they taught. In contrast, role-play quality was the most uniform dimension (CV=5.7%), with all agents sustaining their assigned personas comparably well. Adaptive difficulty control was low across nearly all agents, emerging as a shared weakness rather than a source of between-agent variation. Thus, the agents were broadly comparable in role enactment but diverged mainly in the knowledge content they delivered.

### Role Paradigms Shaped Dimension Profiles, but Empathy Did Not Ensure Knowledge Coverage

**Figure 4.**
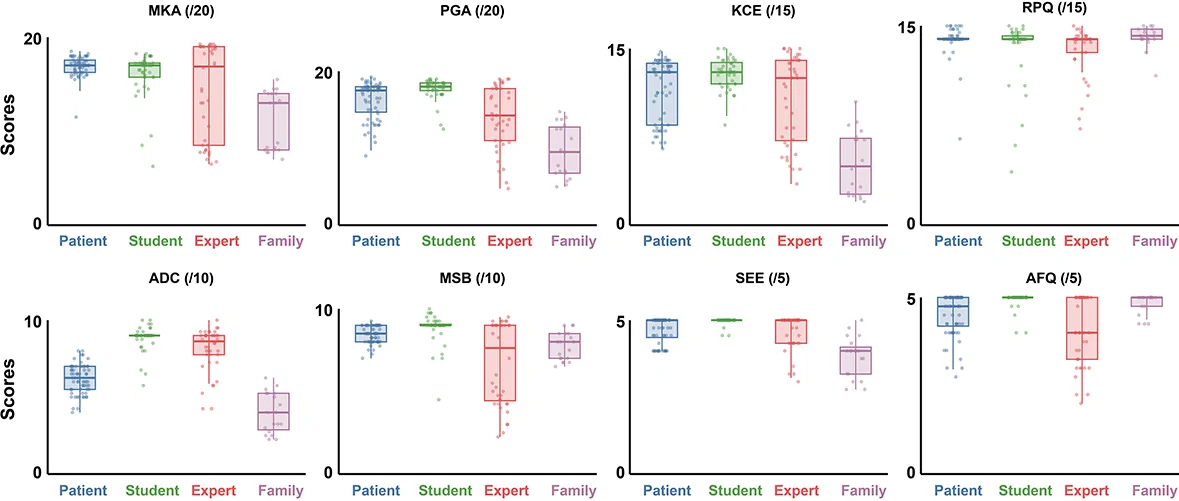
Eight-dimension score distributions across the 4 role paradigms: patient (A1, A4, A5; n=64 dialogues), student (A2, A3; n=44), expert (A6, A7; n=40), and family member (A8; n=19). Each panel shows 1 dimension (scored out of its maximum, indicated in parentheses). Boxes show median and IQR; points are individual dialogue scores. Between-group differences are descriptive; formal inferential testing was not performed at the agent level. ADC: adaptive difficulty calibration; AFQ: assessment and feedback quality; KCE: knowledge-point coverage efficiency; MKA: medical knowledge accuracy; MSB: medical safety boundary; PGA: pedagogical guidance ability; RPQ: role-play quality; SEE: student engagement elicitation.

Grouping agents by role paradigm revealed distinct dimension profiles (Figure 4). At the group level, student-type agents showed the strongest pedagogical guidance (group-mean pedagogical guidance ability was 17.7), whereas expert-type agents showed the lowest medical safety (group-mean medical safety boundary was 6.7). The family-member agent illustrated a notable dissociation between empathy and knowledge: it attained one of the highest role-play scores (role-play quality=14.1) yet the lowest knowledge-point coverage of all agents (knowledge-point coverage efficiency=5.3) and the lowest total score (60.6). Notably, this agent had been deliberately revised after the first 7 agents showed generally limited empathic engagement in student use; only its prompt was adjusted—while all other settings were held constant—to strengthen the empathic, family-member persona. Despite this targeted enhancement of empathy, it remained weakest in knowledge coverage, indicating that strengthening empathy did not translate into broader knowledge delivery. How to balance affective engagement and knowledge coverage within a single agent thus remains an open question. Because each role group comprised only 2 to 3 agents, these between-group comparisons are descriptive; formal inferential testing was not performed at the agent level.

### Student Gender Showed No Detectable Effect on Teaching Quality Scores

Male (n=7) and female (n=15) students were compared across all 8 dimensions using Mann-Whitney *U* tests. No significant difference was detected in any dimension or in total score (79.1 vs 80.1; *P*=.82; all dimension-level *P*>.05; Cohen *d*=0.07 for total score). Given the small, imbalanced sample and the very small observed effect size, this analysis was substantially underpowered; the absence of a detectable difference should therefore not be interpreted as evidence of gender-equitable delivery, as a type II error cannot be excluded.

### AI Scores Agreed With Expert Ratings on Overall and Cognitive-Process Dimensions

**Figure 5.**
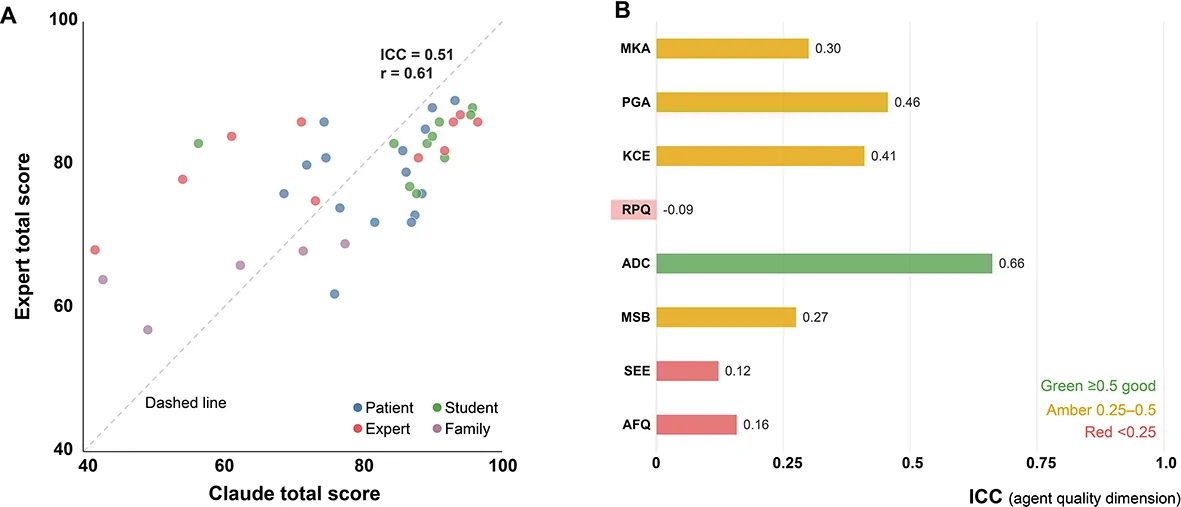
Agreement between Claude AI and expert ratings on 40 dialogues, independently scored by a medical-education expert using the same 8-dimension rubric. (A) Total score agreement: expert versus Claude total scores (the dashed line indicates perfect agreement). (B) Per-dimension agreement represented by intraclass correlation coefficient (ICC) per dimension (green≥0.5; amber 0.25-0.5; red<0.25). ADC: adaptive difficulty calibration; AFQ: assessment and feedback quality; KCE: knowledge-point coverage efficiency; MKA: medical knowledge accuracy; MSB: medical safety boundary; PGA: pedagogical guidance ability; RPQ: role-play quality; SEE: student engagement elicitation.

To validate the rubric-based AI scoring, 40 dialogues spanning all 8 agents and the full quality range were independently rescored by a medical-education expert using an identical rubric, blinded to the AI scores (Figure 5A). AI and expert ratings were closely aligned in magnitude (mean totals 79.1 vs 78.5) and were positively correlated at the total-score level (*r*=0.61; ICC=0.51). Because the two derive from a highly consistent automated rater and a single subjective human expert, close but not exact agreement was expected; the aim was to confirm convergence on the same construct, not identity.

Agreement was strongest on the cognitive-process dimensions, ranging from moderate for knowledge accuracy to highest for adaptive difficulty control. For role-play quality and the two 5-point dimensions, the ICC was near zero or negative, but this reflects a ceiling effect rather than rater disagreement: both AI and expert rated these dimensions uniformly high (for role-play quality, 13.4 vs 13.3 out of 15), leaving minimal between-agent variance (Figure 5B). Because the ICC indexes relative discrimination, it is driven toward zero when there is little variance to rank, and its low value here does not indicate that the raters disagreed; on the contrary, they consistently converged on high scores [24]. The moderate agreement on knowledge accuracy likewise does not undermine the study’s key knowledge-related finding, which rests on a rank-level difference too large to depend on rating precision: A8 scored markedly lowest on knowledge coverage under both AI and expert scoring.

### Platform and Examination Performance Captured Constructs Distinct From Rubric-Based Agent Quality

**Figure 6.**
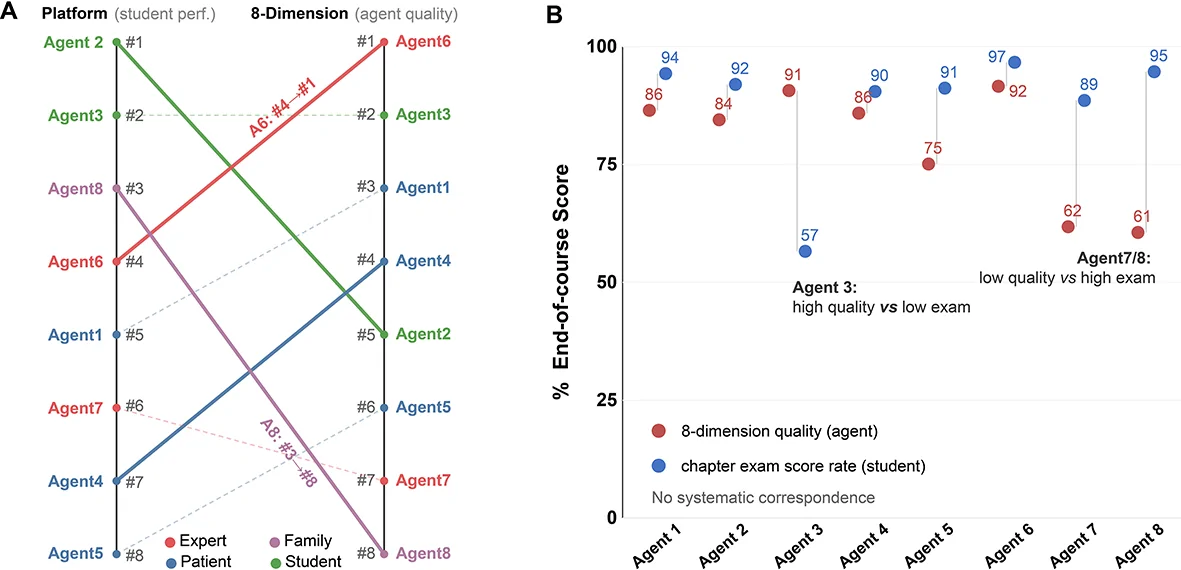
Platform and examination performance versus rubric-based agent quality. (A) Rank divergence: agent rankings by platform score (student performance) vs by 8-dimension quality (agent quality); 1=best. (B) Eight-dimension agent quality versus end-of-course chapter examination score rate.

Agent rankings from platform scores and from the 8-dimension rubric diverged substantially (Figure 6A). Two agents illustrate this most clearly: A8 ranked third by platform score but last by rubric quality, and A6 rose from fourth to first. Rather than indicating that either measure is invalid, this divergence reflects that the two capture different constructs—platform scores index student performance during the interaction, whereas the rubric indexes the teaching quality of the agent itself. The two are therefore complementary rather than interchangeable.

There was a similar lack of association between rubric quality and end-of-course chapter examination performance (Figure 6B): higher agent quality did not correspond to higher chapter examination scores (eg, A3 showed high quality but the lowest examination score rate, whereas A7 and A8 showed low overall quality but high examination scores). This absence of a systematic association should be interpreted cautiously, given the small number of agents, the near-ceiling examination scores, and the lack of individual baseline data; it indicates only that agent teaching quality and this particular examination did not track one another, not that better teaching is ineffective.

## Discussion

This study set out to test whether platform-generated scores, the default metric for AI teaching agents in many educational platforms, adequately capture teaching quality, and to ask what a transparent, multidimensional alternative would reveal. The findings speak less to any single agent than to how the field should evaluate, and design, conversational AI tutors.

Evaluation is not a solved problem, and the default metric may mislead. The most consequential implication is that the metric an educator happens to have at hand can point in the wrong direction. When a platform score and a criterion-based rubric rank the same agents in nearly opposite orders, the choice of metric is not a technical detail but a determinant of which agents get adopted, refined, or discarded. Crucially, this does not make either metric wrong. A platform score that reflects student performance during an interaction and a rubric that reflects the agent’s teaching behavior are answering different questions; the problem arises only when one is silently substituted for the other. As AI tutors proliferate faster than the frameworks to evaluate them [11,14], this argues for treating evaluation itself as a first-class research object, reporting what a metric measures, and pairing convenient platform analytics with transparent, education-specific criteria rather than trusting either alone [17].

Automated evaluation is promising but must be bounded. Using LLMs to score pedagogical quality is attractive because it scales, but our results caution against naive trust [25,26]. Independently developed models converged on the ordering of agents yet differed markedly in leniency, showing that agreement on ranking can coexist with disagreement on absolute standards, and that some models are too lenient to discriminate at all [13,23]. Human calibration therefore remains indispensable [27], and it is not uniform across constructs: automated and expert judgments aligned well on cognitive-process dimensions but poorly on more subjective, affective ones [27]. This suggests a pragmatic division of labor, in which automated scoring is trusted most for the dimensions where it is demonstrably calibrated, while human judgment is reserved for those where meaning is contested [12].

Designing for the whole of teaching, not its most visible parts, and comparing role paradigms, rather than the field’s default patient-doctor script [8,9], exposed a design tension that is easy to miss when engagement is the headline goal. An agent optimized to be more empathic became more engaging without becoming more knowledgeable, a reminder that affective and cognitive teaching functions do not automatically move together [17], and that improving the salient, likable qualities of a tutor can leave its instructional substance untouched. Rather than seeking a single best role, this points toward designing for complementarity, orchestrating different pedagogical strengths across an interaction [16], and toward evaluation that can detect when a more engaging agent is not in fact teaching more. The most uniform finding across every agent, namely persistent weakness in adapting difficulty and providing formative feedback, reinforces the same lesson: the harder and less visible pedagogical skills are precisely those that current prompt-based designs deliver least well [9,28].

Future directions should consider 3 points. First, disentangling role from content requires a factorial design that crosses role paradigms with content domains, so that the effect of how an agent teaches can be separated from what it teaches [5]. Second, establishing educational value, rather than teaching quality alone, requires learning-outcome designs with pre- and postlearning knowledge assessment and individual-level linkage [10,19], ideally using more discriminating assessments than a near-ceiling course examination. Third, the persistent deficits in adaptive difficulty and feedback motivate designs that move beyond static prompting toward dynamic learner modeling and memory-augmented tutoring [8,16], evaluated against the dimensions on which current agents underperform. More broadly, the 8-dimension evaluation framework introduced here offers a transferable reference for the development of future teaching agents, and of medical teaching agents in particular, by making explicit the pedagogical dimensions against which such agents should be designed and assessed.

Undeniably, this is a small, single-institution study in one discipline, and its design confounds role paradigm with content domain, each agent being one role and one chapter, so role-related observations are descriptive rather than causal and no independent content-domain effect can be claimed. Because role groups contain few agents, group comparisons are descriptive and were not tested inferentially at the agent level. Expert calibration rested on a single expert, precluding expert-to-expert reliability, and was weaker for subjective dimensions. The gender comparison was underpowered and cannot support claims of equitable delivery, the rubric weights were team-assigned without formal stakeholder input, and teaching quality rather than learning gain was the outcome assessed. These constraints frame the study as an exploratory, framework-building effort whose specific estimates await confirmation in larger, factorial, outcome-linked designs.

Platform scores answer a different question than teaching quality and should complement rather than replace criterion-based evaluation. By making the dimensions of teaching explicit, and by validating those judgments against expert and cross-model agreement, a multidimensional rubric reveals distinctions that convenient metrics miss, including where more engaging agents are not more instructive and where all current agents falter. Beyond any single result, the study suggests that how we evaluate AI teaching agents is inseparable from how we improve them.

## Supplementary material

10.2196/96819 Multimedia Appendix 1 Complete configuration of the 8 AI teaching agents.

10.2196/96819 Multimedia Appendix 2 The complete 8-dimension scoring prompt used by all evaluators.

## Abbreviations

CV: coefficient of variation
ICC: intraclass correlation coefficient
LLM: large language model
