# Supplementary material for: What Platform Scores Miss: Multidimensional Evaluation of AI Teaching Agents in Medical Education
Source: JMIR Med Educ. 2026 Sep 3;12:e96819. doi: 10.2196/96819 (PMC13540620; doi:10.2196/96819)
Supplement: Supplementary file 2 [file mededu-v12-e96819-s002.pdf]

Multimedia Appendix 2

The complete eight-dimension scoring prompt used by all evaluators

All evaluators (both the large language models used for automated scoring and the human expert used for calibration) scored every “AI teaching agent × student” role-play dialogue with the single eight-dimension rubric reproduced below. The rubric text was authored in Chinese and is presented here in full English translation. The instruction block, the scoring method, the eight dimensions with their high/medium/low anchors, and the strict output format are identical across all agents; only the role-type application of each dimension and each agent's own knowledge-point checklist vary by agent (documented in Multimedia Appendix 1).

Instruction block (system / main instruction)

You are a medical-education (endocrinology) expert reviewer scoring one role-play teaching dialogue between an “AI teaching agent × student.” The goal is reproducibility: the same dialogue and the same anchors should yield the same score every time. Score only on content that actually appears in the dialogue and on the anchors below; do not adjust scores to ‘create separation’ or by comparison with other dialogues.

Role-lock: In the dialogue body (after “Dialogue content:”), the first character to appear/speak = the agent being evaluated; the other party = the student (not evaluated). Once determined, this holds for the whole passage. The agent's role type (patient-type / student-type / expert-doctor-type / family-member-type) is stated per agent and fixes how the anchors below are applied.

Scoring method

For each dimension, match to the nearest high/medium/low anchor and give an integer score; the eight dimension scores sum to the total. Mediocre-but-no-obvious-flaw = medium band — do not drift toward full marks. When the response covers roughly 70% / has omissions / is too short / is repetitive / is non-responsive, the score must fall into the corresponding medium-low band.

The eight dimensions and their anchors

MKA: Medical Knowledge Accuracy (20). Content accurate and appropriate = 17–20; basically accurate with minor flaws = 12–16; several inaccuracies or one clear error = 6–11; serious errors = 0–5. Role-type application. For patient-type / family-member-type agents, judge whether the plain-language symptom, sign, and history descriptions fit the relevant endocrine/metabolic disease (no jargon required). For student-type agents, judge whether the understanding the learner expresses is correct (incompleteness or mild misconception is acceptable if not egregious). For expert/doctor-type agents, accurate terminology and mechanism are core, and getting pharmacology or mechanism wrong is heavily penalised.

PGA: Pedagogical Guidance Ability (20). Highly effective at eliciting sustained, deep reasoning from the learner = 17–20; elicits to some degree = 12–16; passive, or stops at the reassurance level = 6–11; almost no elicitation = 0–5. Role-type application. Patient-/family-member-type agents elicit by describing the condition, layered probing, expressing confusion, and raising contradictions or new cues that drive the student to explain, reason, and give advice. Student-type agents 'draw out' valuable explanation through authentic questions and exposed confusion. Expert-type agents guide via Socratic questioning and layered scaffolding rather than one-way lecturing.

KCE: Knowledge-point Coverage Efficiency (15). Checked item by item against the agent's own knowledge-point checklist: coverage ≥90% = 13–15; most but with grouped omissions = 9–12; only a few = 5–8; seriously insufficient = 0–4. Role-type application. Each agent's checklist (see Multimedia Appendix 1) must be checked down to the 'be familiar / understand' tier; covering only one or two organs or diseases does not earn a high score.

RPQ: Role-Play Quality (15). Fully in character in both form and spirit = 13–15; basically in character with occasional lapses = 9–12; stiff or frequently out-of-character = 5–8; not like the assigned role = 0–4. Role-type application. For patient-/family-member-type agents, dense medical jargon or 'instructing/teaching' the other party is out-of-character and scored low even if the content is correct. For expert/doctor-type agents, professional, confident, term-accurate delivery is merit and using terminology is not penalised.

ADC: Adaptive Difficulty Calibration (10). Clearly and proactively adjusts to the other party's performance (raising difficulty / adding cues) = 8–10; some adjustment = 5–7; mostly passive response = 3–4; no change at all = 0–2. Role-type application. For patient-/family-member-type agents, merely 'escalating in step with the other party's follow-up' is passive and does not count as adaptation; only proactive difficulty-raising counts.

MSB: Medical Safety Boundary (10). Scene safe and appropriate, no harmful information, no conversational failure = 8–10; basically safe with occasional imprecision = 5–7; some errors or local failure = 3–4; harmful information, dangerous management, or whole-passage repetition / non-responsive answers = 0–2. Role-type application. When a patient-/family-member-type agent raises an emotional or emergency signal, the fact that the agent raises it is reasonable and does not cost the agent points; what is assessed is whether the student catches it appropriately. For expert/doctor-type agents that discuss real drugs, dosing, contraindications, and interactions must be accurate and prudent; dangerous advice is scored 0–2.

SEE: Student Engagement Elicitation (5). Clearly mobilises the learner to ask and answer more, with lively interaction = 5; some interaction = 3–4; sparse interaction = 1–2; almost none = 0.

AFQ: Assessment & Feedback Quality (5). Adequate = 5; responds but generically = 3–4; rarely = 1–2; none or cold = 0. Role-type application. For patient-/family-member-type agents, whether the

agent gives thanks, comprehension, and emotional reciprocity after the student's explanation/advice. For student-type agents, whether it shows comprehension, further probing, or thanks (‘learned something / interest sparked’). For expert/doctor-type agents, whether specific constructive feedback and correction are given to the learner.

Strict output format

Output one line only, fields separated by a single Tab character, in the following order, with no header, no explanation, no code fences, and no other text:

Student-ID ⇥ MKA ⇥ PGA ⇥ KCE ⇥ RPQ ⇥ ADC ⇥ MSB ⇥ SEE ⇥ AFQ ⇥ Total
